# UCP2-induced hypoxia promotes lipid accumulation and tubulointerstitial fibrosis during ischemic kidney injury

**DOI:** 10.1038/s41419-019-2219-4

**Published:** 2020-01-13

**Authors:** Qingqing Ke, Qi Yuan, Nan Qin, Caifeng Shi, Jing Luo, Yi Fang, Lingling Xu, Qi Sun, Ke Zen, Lei Jiang, Yang Zhou, Junwei Yang

**Affiliations:** 1 Center for Kidney Disease, Second Affiliated Hospital, Nanjing Medical University, Nanjing, China; 2 State Key Laboratory of Pharmaceutical Biotechnology, Nanjing University Advanced Institute of Life Sciences, Nanjing, China

**Keywords:** Mechanisms of disease, End-stage renal disease

## Abstract

Mitochondrial dysfunction leads to loss of renal function and structure; however, the precise mechanisms by which mitochondrial function can regulate renal fibrosis remain unclear. Proximal tubular cells (PTCs) prefer fatty acid oxidation as their energy source and dysregulation of lipid metabolism has been linked to tubulointerstitial fibrosis (TIF). Here, we demonstrated that mitochondrial uncoupling protein 2 (UCP2) regulates TIF through the stimulation of lipid deposition and extracellular matrix (ECM) accumulation. We show that UCP2 expression was increased in human biopsy sample and mouse kidney tissues with TIF. Moreover, UCP2-deficient mice displayed mitigated renal fibrosis in I/R-induced mouse model of TIF. Consistent with these results, UCP2 deficiency displayed reduced lipid deposition and ECM accumulation in vivo and in vitro. In UCP2-deficient PTCs, inhibition of TIF resulted from downregulation of hypoxia-inducible factor-1α (HIF-1α), a key regulator of lipid metabolism and ECM accumulation. Furthermore, we describe a molecular mechanism by which UCP2 regulates HIF-1α stabilization through regulation of mitochondrial respiration and tissue hypoxia during TIF. HIF-1α inhibition by siRNA suppressed lipid and ECM accumulation by restoration of PPARα and CPT1α, as well as suppression of fibronectin and collagen I expression in PTCs. In conclusion, our results suggest that UCP2 regulates TIF by inducing the HIF-1α stabilization pathway in tubular cells. These results identify UCP2 as a potential therapeutic target in treating chronic renal fibrosis.

## Introduction

Mitochondrial damage or dysfunction contributes critically to the pathogenesis of various kidney diseases, including acute kidney injury and chronic kidney disease (CKD)^1–3^. A critical function of mitochondria is to provide energy that is used by the kidney to remove waste products from the blood as well as to regulate fluid and electrolyte balance. The provision of energy to the cell is through the electron transport chain located in the inner membrane of mitochondria in a process called OXPHOS. Uncoupling proteins (UCPs), also located in the inner membrane is a superfamily of mitochondrial anion carrier proteins that uncouple OXPHOS from ATP synthesis with energy dissipated as heat^4–6^. Although with weak uncoupling activity, UCP2 is previously identified in regulating cellular energy homeostasis in various cell types^7–16^. Moreover, UCP2 affects the expression of lipid regulatory enzymes and transcription factors in mice^11,14^. Our recent studies suggest that UCP2 regulates cytoskeleton, autophagy, and extracellular matrix (ECM) production in kidney disease^17^. Furthermore, we have shown that UCP2 modulates mitochondrial membrane potential and is vital for mitochondrial dynamics^18^.

Emerging evidence suggests the involvement of lipid in the pathogenesis of tubulointerstitial fibrosis (TIF). Under normal conditions, β-oxidation of fatty acid is the main energy substrates and preferable energy source in proximal tubular cells (PTCs). However, lipid accumulation resulted from dysfunction in lipid utilization is toxic to tubular cells and leads to ECM production and renal fibrosis^19^. Recent studies suggested that lipid accumulation is tightly regulated by various key enzymes and regulators, which warrants further investigation. The integration of lipid derangement, mitochondrial dysfunction, and ECM production and their contribution to the pathogenesis of renal fibrosis remain incompletely understood. We hypothesized that metabolic regulation by mitochondria may represent a critical determinant of the pathogenesis of TIF. We therefore sought to study the effect of metabolic regulation on lipid by modulating the expression of mitochondrial UCP2.

Hypoxia has been proposed as an important microenvironmental factor in the development of kidney fibrosis^20–22^. Clinical and genetic evidence shows that activation of hypoxia-inducible factor-1 (HIF-1) in tubular cells is associated with the development of CKD and promotes fibrogenesis by increasing ECM accumulation^23^. Moreover, recent studies suggest that HIF-1α is a critical transcription factor in regulating lipid metabolism^24–26^. The role of UCP2 on tissue hypoxia during TIF currently remains unknown. We hypothesized that modulation of hypoxia and HIF-1α by UCP2 regulates renal fibrosis through promoting lipid and ECM accumulation.

In the current study, we show that UCP2 expression was increased in human biopsy sample with TIF. UCP2-deficient mice displayed mitigated renal fibrosis in ischemia/reperfusion injury (I/R)-induced mouse model of TIF. Consistent with these results, UCP2 deficiency displayed reduced lipid deposition and ECM accumulation in vivo and in vitro. Importantly, we demonstrated that UCP2 regulates lipid and ECM by regulating of HIF-1α in PTCs. We describe a molecular mechanism by which UCP2 regulates HIF-1α stabilization through regulation of mitochondrial respiration and tissue hypoxia during TIF. Moreover, the genetic inhibition of HIF-1α suppressed lipid and ECM accumulation by restoration of PPARα and CPT1α, as well as suppression of fibronectin and collagen I expression. Our results suggest that UCP2 is a propathogenic mediator in TIF through the regulation of HIF-1α stabilization in tubular cells.

## Materials and methods

### Animals

Our study was approved by the Ethical Commission of Nanjing Medical University (Nanjing, Jiangsu Province, China). Animals were treated humanely according to guidelines of the Institutional Animal Use and Care Committee at the Nanjing Medical University. Male C57BL/6J mice were purchased from Shanghai experimental animal center (Shanghai, China) and were housed in the animal facilities of the experimental animal center of Nanjing Medical University with free access to food and water. Transgenic B6.Cg-Tg(Ggt1-cre)M3Egn/J mice that express cre under the control of *Ggt1* promoter were purchased from The Jackson Laboratory. The GeneBank Accession Number for UCP2 is NM_011671.4. C57BL/6J embryonic stem cells were used for gene targeting. The targeting strategy allows the generation of a conditional knockout (KO) mUcp2 allele; we identified eight exons, with the ATG start codon in exon 3 and TGA stop codon in exon 8; exon 3 and exon 4 were selected as conditional KO region. Deletion of exon 3 and exon 4 should result in the loss of function of the mUcp2 gene; to engineer the targeting vector, 5′ homology arm, 3′ homology arm, and condition KO (CKO) region will be amplified from BAC DNA and confirmed by end sequencing; in the targeting vector, the Neo cassette was flanked by Frt sites, and CKO region was flanked by LoxP sites. Diptheria toxin A (DTA) was used for negative selection. The constitutive KO allele was obtained after cre-mediated recombination. Primers used for genotyping were as follows: UCP2_F1: TGG AAT TCA TCA AGG TGT CTC ATG TC; UCP2_F2: ACT GGG CCA GAA GCA CAA TGG; UCP2_R2: CCC AGC TCT ACT TCT CCC TGG AGA; cre Primer F: GAA CGC ACT GAT TTC GAC CA; cre Primer R: GCT AAC CAG CGT TTT CGT TC.

Mouse models of TIF were induced using I/R, folic acid nephropathy (FAN) and aristolochic acid nephropathy (AAN). Mice aged ~8 weeks (~22 g) were randomly assigned into different groups with at least seven mice per group: sham, 6 weeks after I/R, 2 weeks after FAN, and 2 weeks after AAN. I/R was performed using an established procedure^27,28^. A pair of microvascular clamps (S&T, Swiss) was applied to both pedicles to block renal perfusion for 30 min. Folic acid (F7876, Sigma-Aldrich) dissolved in 300 mmol/L NaHCO_3_ was once injected intraperitoneally at a dose of 250 mg/kg. Aristolochic acid I sodium salt (A9451, Sigma-Aldrich) was daily administered intraperitoneally at a dose of 2.5 mg/kg. Same volume of saline with adjusted pH value was administered in control mice. Blood and kidney samples were harvested for further analysis. No blinding was done.

### Cell culture and treatment

Primary PTCs were cultured under sterile conditions from collagenase-digested cortical fragments of kidneys isolated from mice (~21 days) by a modification of previously described methods^29^. Briefly, renal cortices were dissected visually in ice-cold dissection solution (DS) and sliced into pieces of ~1 mm wide. The fragments were transferred to collagenase solution at 37 °C and digested for 30 min. After digestion, the supernatant was sieved through two nylon sieves (pore size 250 and 80 μm) to yield a large number of long proximal tubule (PT) fragments (~100 μm in length) without substantial contamination of other nephron segments or glomeruli. The longer PT fragments were resuspended by flushing the sieve in the reverse direction with warm DS (37 °C) containing 1% (wt/vol) bovine serum albumin (BSA) and then centrifuged for 5 min at 170 × g, washed, and resuspended into the appropriate amount of culture medium. The PT fragments were seeded onto collagen-coated permeable PTFE-filter supports and left unstirred for 48 h at 37 °C and 5% CO_2_ in a standard humidified incubator, after which the culture medium was changed for the first time. The medium was then replaced every 2 days. After 7 days, cell cultures were organized as a confluent monolayer.

For hypoxia/reoxygenation (H/R) treatment, cells were placed in hypoxic conditions, which were a sealed chamber filled with 93% N_2_, 5% CO_2_, and 2% O_2_ (STEMCELL Technologies Inc., Vancouver, BC, Canada) at 37 °C for 12 h and then placed in normoxia conditions for another 12 h. The oxygen concentration was checked at the beginning and end of the hypoxic period by an oxygen analyzer. Control cells were kept in normoxia conditions (21% O_2_ and 5% CO_2_) at 37 °C for the same time periods.

PTCs were transiently transfected with negative control (N.C.) short interfering RNA (siRNA) or HIF-1α siRNA (Ibsbio, Shanghai, China) using lipofectamine RNAiMAX transfection reagent (13778, Invitrogen) according to the manufacturer’s instructions. After transfection for 24 h, PTCs were then exposed to the H/R. The sequences of siRNA were as follows: HIF-1α: sense 5′-GAU GGA AGC ACU AGA CAA AGU-3′; anti-sense 5′-UUU GUC UAG UGC UUC CAU CAG-3′. N.C.: sense 5′-UUC UCC GAA CGU GUC ACG UTT-3′; anti-sense 5′-ACG UGA CAC GUU CGG AGA ATT-3′.

### Measurement of OCR

A Seahorse Bioscience X24 extracellular flux analyzer was used to measure the rate change of dissolved O_2_ in medium immediately surrounding adherent cells cultured in an XF24 V7 cell culture microplate (Seahorse Bioscience). Mouse primarily cultured tubular cells were seeded in a XF24 V7 cell culture microplant at a 1.0 × 10^4^ cells per well. Oxygen consumption rate (OCR) (pmol/min) was assessed at baseline and after the addition of oligomycin (1 μmol/L), followed by the addition of Carbonyl cyanide 4-(trifluoromethoxy) phenylhydrazone (FCCP) (0.75 μmol/L). Final state was determined after the addition of the ATP synthase inhibitor oligomycin or rotenone (1 μmol/L). Finally, OCR (pmol/min/μg protein) was normalized by protein concentration.

### Renal function analysis

Renal function was evaluated by level of blood urea nitrogen (BUN) and serum creatinine (Scr) measured with the QuantiChrom Urea and Creatinine Assay kit (DIUR-500, DICT-500, Hayward, CA) according to the manufacturer’s instructions, respectively.

### Histology assay

Neutraformalin (10%)-fixed kidney samples were kept at 4 °C overnight. The samples were then paraffin embedded and sectioned at 3 μm in thickness for hematoxylin and eosin (H&E), masson, or sirius red staining. Slides were viewed with a Nikon Eclipse 80i microscope equipped with a digital camera (DS-Ri1, Nikon, Shanghai, China). The percentage of interstitial fibrotic area to the selected field was analyzed using Image Pro Plus 6.0 software. An average percentage of kidney fibrotic area for each section was calculated. At least ten randomly chosen fields under the microscope were evaluated for each mouse, and an average score was calculated.

### Total collagen content determination

Total collagen content within kidney tissue was quantitated as reported previously, and the ratio of collagen to total protein was expressed as micrograms per milligram of total protein^30^.

### Western immunoblot analysis

Western blot was performed as previous described^31^. The primary antibodies used were as follows: anti-UCP2 (sc-6526, Santa Cruz), anti-PPARα (ab24509, Abcam, Cambridge, MA, US), anti-CPT1α (ab128568, Abcam), anti-fibronectin (F3648, Sigma Aldrich), anti-collagen I (1310–01, Southern Biotech), anti-HIF-1α (ab1, Abcam), anti-PHD2 (4835, Cell Signaling Technology), anti-VDAC (ab14734, Abcam), and anti-Tubulin (T6074, Sigma Aldrich). Western blot were performed at least three times independently. Quantification was performed by measurement of the intensity of the signals with the aid of National Institutes of Health Image software package.

### Quantitative polymerase chain reaction analysis

Quantitative polymerase chain reaction (Q-PCR) was performed using an Applied Biosystems 7300 Sequence Detection system. Total RNA of tissues was prepared using a TRIzol isolation system according to the instructions by the manufacturer (Invitrogen). The first strand of cDNA and subsequent real-time quantification was performed according to the instructions by the manufacturer (Thermofisher Scientific). All reactions were run in triplicate. The cycle threshold (CT) data were determined using default threshold settings and the mean CT was calculated from the triplicate PCRs. The ratio of mRNA was calculated by using the equation 2^−ΔCT^, in which ΔCT = CT_treatment_ − CT_control_. All primers were purchased from Invitrogen. Actin was used for normalization in mRNA Q-PCR when total RNA was extracted from tissue samples.

### Immunohistochemical staining

Immunohistochemical staining of kidney sections was performed using paraffin-embedded sections. In brief, they were stained with UCP2 antibody (sc-6527; Santa Cruz Biotechnology), PPARα antibody (ab24509, Abcam, Cambridge, MA, US), CPT1α antibody (ab128568, Abcam), fibronectin antibody (F3648, Sigma Aldrich), or collagen I antibody (1310–01, Southern Biotech) using the Vector Mouse on Mouse (M.O.M.) immunodetection Kit, according to the manufacturer’s protocol (Vector Laboratories, Burlingame, CA). Slides were viewed with a Nikon Eclipse E80i microscope equipped with a digital camera (DS-Ri1; Nikon).

### Mitochondrial morphology and immunofluorescent staining

Mitochondria in cells cultured on cover slips were labeled by incubating living cells with the fluorescent probe MitoTracker Red (250 nM for 30 min, Molecular Probes, Invitrogen). Then cells were washed, fixed, blocked, and then incubated with the specific primary antibody: anti-CPT1α (ab128568, Abcam), anti-HIF-1α (ab1, Abcam), anti-fibronectin (F3648, Sigma Aldrich), or anti-collagen I (1310–01, Southern Biotech) followed by staining with FITC-conjugated secondary antibody. Cells were double stained with 40, 6-diamidino-2-phenylindole (DAPI) to visualize the nuclei. Slides were viewed with a confocal inverted laser microscope (LAM 510 Meta, Zeiss).

### Lipid droplets staining

Freshly prepared kidney tissues were OCT-embedded and sectioned at 12 μm for Oil red O staining and 3 μm for BODIPY (D3922, Thermo Fisher, US) staining. After rinse with isopropanol, slides were stained with freshly prepared Oil red O (Sigma-Aldrich, US) working solution according to the manufacturer’s instructions. The nuclei were lightly stained with alum hematoxylin. After stained with BODIPY, slides were stained with immunostained with laminin (L9393, Sigma-Aldrich) and DAPI. Slides were viewed with a Nikon Eclipse 80i microscope equipped with a digital camera (DS-Ri1, Nikon, Shanghai, China). The percentage of positive area to the selected field was analyzed using Image Pro Plus 6.0 software. An average positive area for each section was calculated. At least ten randomly chosen fields under the microscope were evaluated for each sample, and an average score was calculated.

### Measurement of hypoxia

Tissue and cell hypoxia were determined using hypoxyprobe^TM^ kit (Hypoxypeobe, Inc. Burlington, MA, US), which contains pimonidazole hydrochloride and anti-pimonidazole mouse IgG_1_ monoclone antibody (MAb1). Pimonidazole is reductively activated in hypoxic cells. The activated intermediate forms stable covalent adducts with thiol (sulfydryl) groups in proteins, peptides, and amino acids. The antibody reagent MAb1 binds to these adducts allowing their detection by immunochemical means. Pimonidazole hydrochloride was intraperitoneally administered at the recommended dosage of 60 mg/kg ~1–1.5 h before been sacrificed. Cells were cultured in the presence of 100 μmol/L pimonidazole hydrochloride for 1–2 h before been harvested. Tissue or cell was fixed in cold acetone for 10 min. Then rinsed and incubated overnight at 4 °C with MAb1 diluted in phosphate-buffered saline (PBS) containing 0.1% BSA and 0.1% Tween 20. The sections are then incubated for 90 min with Cy-3 or TRITC-conjugated goat anti-mouse antibody 1:150 (Jackson Immuno Research Laboratories). Between all steps of the staining procedure, the sections are rinsed three times for 2 min in PBS.

### Human kidney tissue handling

The human study protocol conformed to the ethical guidelines of the 1975 Declaration of Helsinki as reflected in a priori approval by the Ethics Committees of Nanjing Medical University for Medical Experiments. Written informed consent was obtained from every enrolled individual. Renal biopsy samples were collected from individuals from the Center for Kidney Disease of Second Affiliated Hospital of Nanjing Medical University.

### Statistical analysis

Data were expressed as mean ± s.e.m. Western blot analysis was completed by scanning and analyzing the intensity of hybridization signals by using NIH Image program. Statistical analysis of data were performed using Sigma Stat software (Jandel Scientific Software, San Rafael, CA). Comparisons between groups were made using one-way analysis of variance (ANOVA), followed by the *t*-test. *p* < 0.05 was considered significant.

## Results

### UCP2 is upregulated in human and mouse renal fibrosis and, promotes I/R-induced TIF

**Fig. 1:**
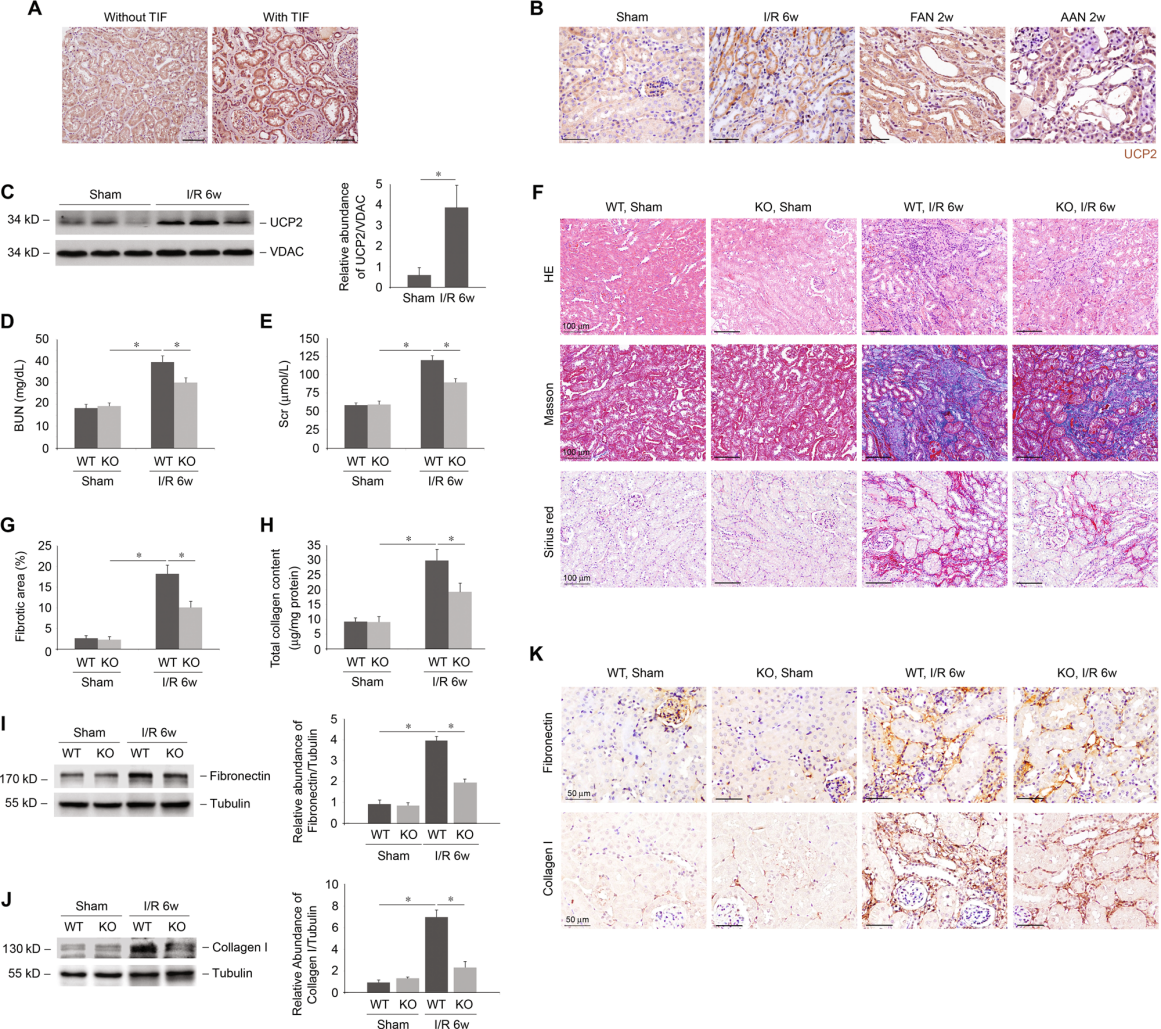
Deficiency of UCP2 protects against I/R-induced TIF. **a** Representative images of human kidney sections with or without tubulointerstitial fibrosis with immunostaining for UCP2. Scale bars, 100 μm. **b** Representative images of mice kidney sections from sham, I/R, FAN, and AAN mice with immunostaining for UCP2. Scale bars, 50 μm. **c** Immunoblot analysis of UCP2 in kidney tissue from mice 6 weeks after I/R or sham laparotomy. Voltage-dependent anion channel (VDAC) served as the standard. *p < 0.05, ANOVA. **d**, **e** Renal dysfunction of I/R was determined in UCP2 wild-type (WT) or knockout (KO) mice 6 weeks after I/R or sham laparotomy. Blood urine nitrogen (BUN) **d** and serum creatinine (Scr) **e** were measured in sera; *n* = 7 per group. *p < 0.05, ANOVA. **f** Representative images of mice kidney sections from UCP2 WT or KO mice 6 weeks after I/R or sham laparotomy with H&E, masson and sirius red staining. Scale bars, 100 μm. **g**, **h** Fibrotic area **g** and total collagen content **h** in the kidneys from UCP2 WT or KO mice 6 weeks after I/R; *n* = 7 per group. *p < 0.05, ANOVA. **i**, **j** Immunoblot analysis of fibronectin **i** and collagen I **j** in kidney tissue from UCP2 WT or KO mice 6 weeks after I/R or sham laparotomy. Tubulin served as the standard. *p < 0.05, ANOVA. **k** Representative images of mice kidney sections from UCP2 WT or KO mice 6 weeks after I/R with immunostaining for fibronectin or collagen I. Scale bars, 50 μm.

We first observed that UCP2 levels in kidney samples from patients with TIF were markedly increased compared with those of control patients without TIF (Fig. 1a). Consistently, the UCP2 levels were markedly increased in kidney samples from mouse models with TIF using I/R, FAN, and AAN, clinically relevant murine models of TIF (Fig. 1b). As compared with FAN and AAN, I/R-induced chronic kidney fibrosis is the best characterized mouse model of chronic TIF without the toxic influence of chemicals. Therefore, we applied I/R model in our subsequent studies. The expression of UCP2 was significantly increased in the kidneys of mice at 6 weeks after I/R relative to that in sham-operated control mice (Fig. 1c). To evaluate the role of UCP2 in TIF induced by I/R, we analyzed the BUN (Fig. 1d) and Scr (Fig. 1e) levels in wild-type (WT) and PTC-specific UCP2 KO mice 6 weeks after I/R relative to those in the corresponding control mice subject to sham surgery. The BUN and Scr were markedly elevated in WT mice after I/R compared with those in sham-operated control mice. In contrast, the BUN and Scr were significantly decreased in KO mice after I/R relative to those in WT mice. Similar to BUN and Scr, renal morphologic changes (Fig. 1f) and TIF (Fig. 1g, h) after I/R were significantly reduced in KO mice compared with those in WT mice. We also analyzed ECM by measurement of expression and deposition of fibronectin and collagen I. We observed that expression of fibronectin (Fig. 1i) and collagen I (Fig. 1j) were increased in WT mice after I/R compared with those in sham-operated control mice, which were accumulated and deposited in tubulointerstitial spaces (Fig. 1k). Consistent with morphologic changes, KO mice displaced reduced ECM relative to WT mice. Taken together, these results suggest that UCP2 is a critical mediator in the pathogenesis of TIF.

### UCP2 deficiency suppresses lipid accumulation

**Fig. 2:**
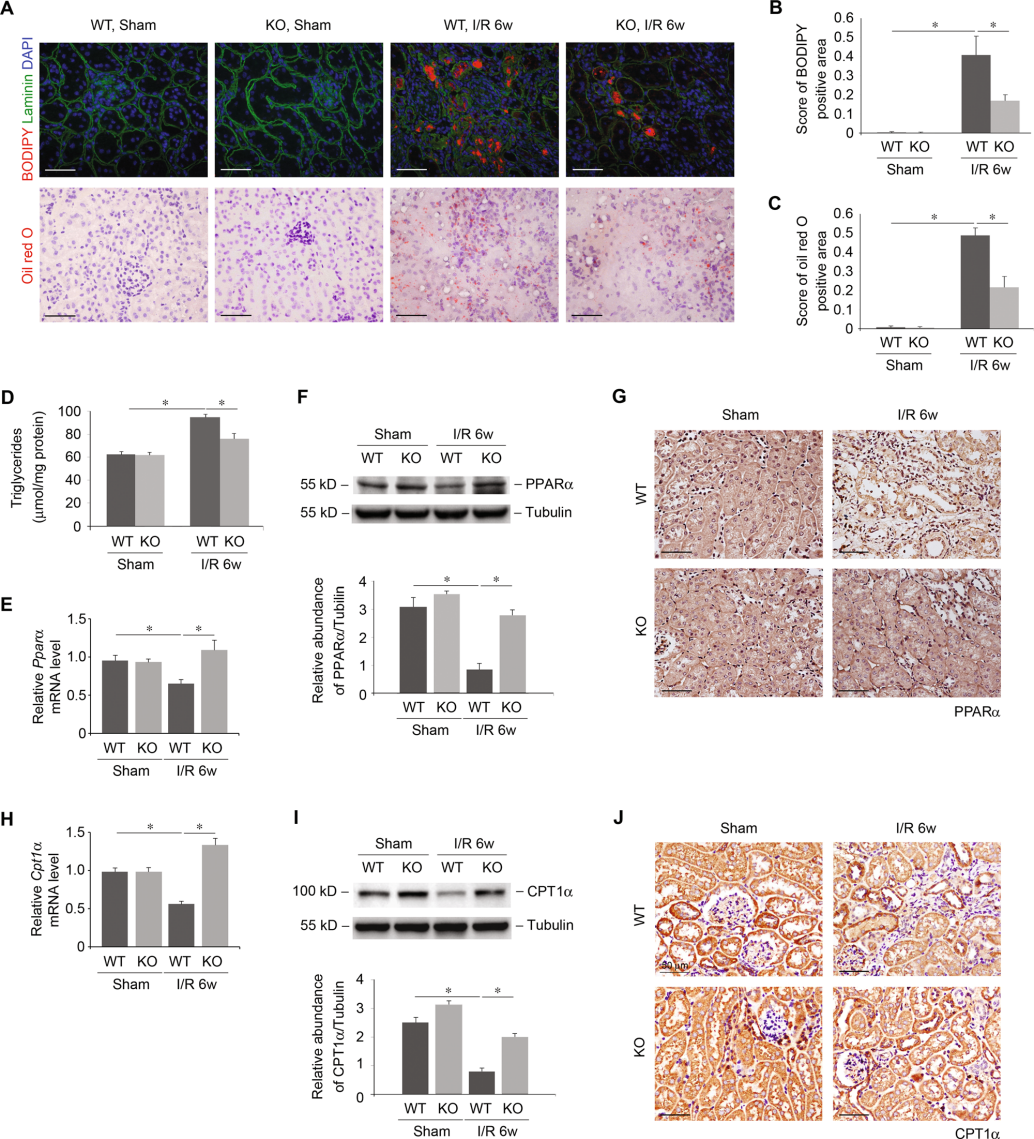
Deficiency of UCP2 inhibits lipid accumulation. **a**–**d** Lipid in kidney tissue was determined in UCP2 WT or KO mice 6 weeks after I/R or sham laparotomy. Representative images of mice kidney sections with bodipy and Oil red O staining **a**, score of bodipy positive area **b**, quantification of Oil red O positive area **c**, and triglycerides contents kidney tissue **d**; *n* = 7 per group. *p < 0.05, ANOVA. Scale bars, 50 μm. **e** Relative mRNA levels of *Pparα* in mice kidney sections from UCP2 WT or KO mice 6 weeks after I/R or sham laparotomy; *n* = 7 per group. *p < 0.05, ANOVA. **f** Immunoblot analysis of PPARα in kidney tissue from UCP2 WT or KO mice 6 weeks after I/R or sham laparotomy. Tubulin served as the standard. *p < 0.05, ANOVA. **g** Re*p*resentative images of mice kidney sections from UCP2 WT or KO mice 6 weeks after I/R with immunostaining for PPARα. Scale bars, 50 μm. **h** Relative mRNA levels of *Cpt1α* in mice kidney sections from UCP2 WT or KO mice 6 weeks after I/R or sham laparotomy; *n* = 7 per group. *p < 0.05, ANOVA. **i** Immunoblot analysis of CPT1α in kidney tissue from UCP2 WT or KO mice 6 weeks after I/R or sham laparotomy. Tubulin served as the standard. *p < 0.05, ANOVA. **j** Representative images of mice kidney sections from UCP2 WT or KO mice 6 weeks after I/R with immunostaining for CPT1α. Scale bars, 50 μm.

The accumulation of lipid droplets has been associated with TIF in humans and mice^19^. Since UCP2 functions in cellular lipid regulation^11,14^, we investigated whether UCP2 can act as a regulator of cellular lipid as it relates to the progression of TIF. We first measured lipid droplets deposition in kidney tissue. Increased lipid droplets deposition was observed in WT mice after I/R compared with those in sham-operated control mice (Fig. 2a). Meanwhile, KO mice displaced reduced lipids relative to WT mice (Fig. 2b, c). Consistently, the level of triglycerides (TGs) was increased in the kidney tissues of WT mice. In contrast, the levels of TGs in kidney tissue were significantly decreased in KO mice relative to those in WT mice (Fig. 2d). Next, we measured PPARα and CPT1α in kidney tissue. mRNA (Fig. 2e) and protein (Fig. 2f) levels of PPARα in tubular cells (Fig. 2g) were markedly decreased in WT mice after I/R compared with those in sham-operated control mice. While KO mice displaced restored expression of PPARα relative to WT mice. Decreased expression of CPT1α in tubular cells of WT mice after I/R were also recovered in those of KO mice (Fig. 2h–j). Together, these results suggest that UCP2 regulates TIF, which was associated with lipid accumulation in vivo.

### UCP2 regulates ECM production and lipid accumulation in primarily cultured PTCs

**Fig. 3:**
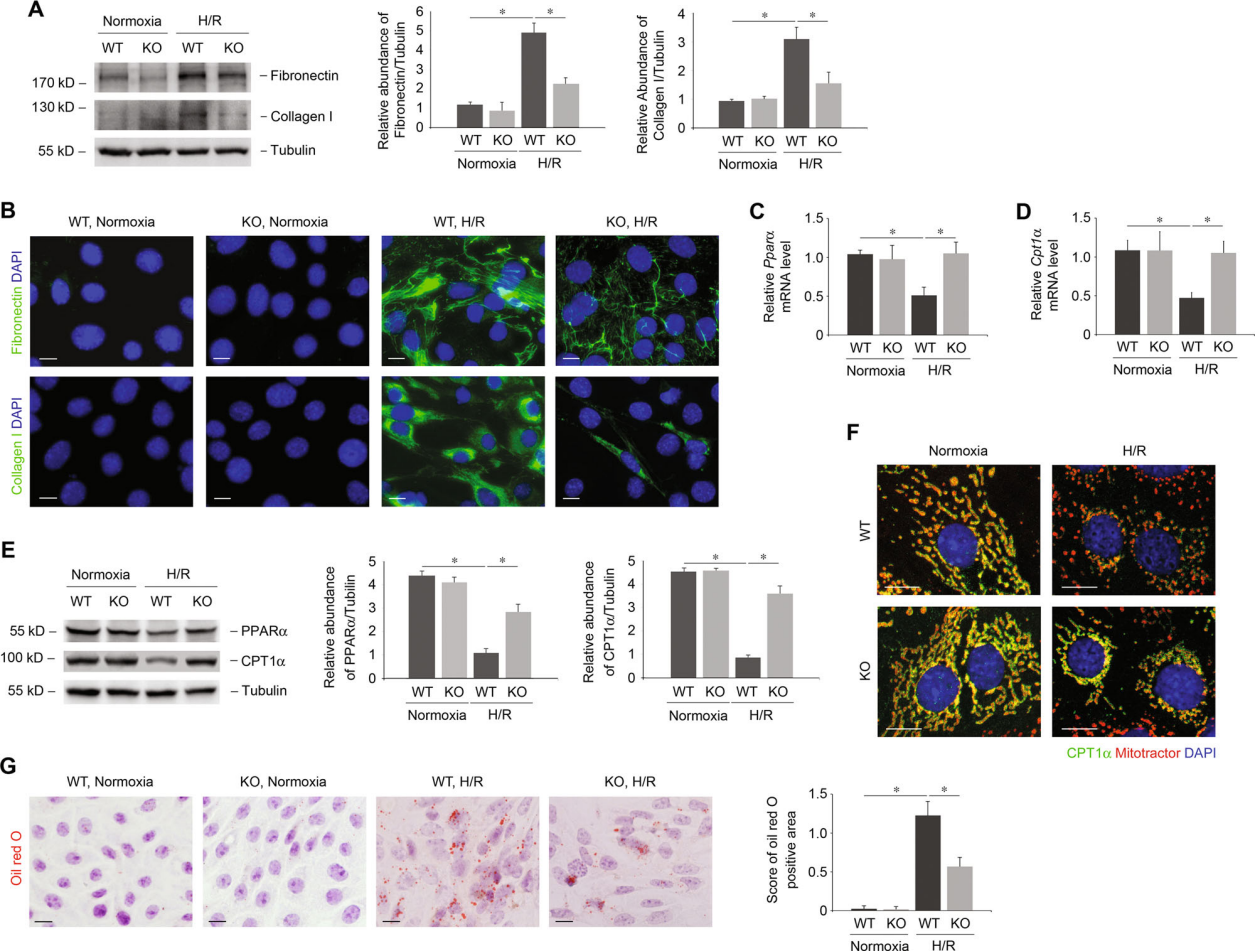
UCP2 regulates ECM production and lipid accumulation in primarily cultured PTCs. **a** Immunoblot analysis for fibronectin or collagen I of cell lysates from UCP2 WT or KO PTCs treated with hypoxia for 12 h, followed by reoxygenation for 12 h (H/R). Tubulin served as the standard. *p < 0.05, ANOVA. **b** Representative images of UCP2 WT or KO PTCs treated with or without H/R with immunostaining for fibronectin or collagen I (green). Nuclei were counterstained with DAPI (blue). Scale bars, 10 μm. **c**, **d** Relative mRNA levels of *Pparα* **c** and *Cpt1α* **d** in UCP2 WT or KO PTCs treated with H/R; *n* = 3 per group. *p < 0.05, ANOVA. **e** Immunoblot analysis for PPARα or CPT1α of cell lysates from UCP2 WT or KO PTCs treated with H/R. Tubulin served as the standard. *p < 0.05, ANOVA. **f** Representative images of UCP2 WT or KO PTCs treated with or without H/R with immunostaining for CPT1α (green). Mitochondrial morphology was visualized by staining with MitoTracker Deep Red (red). Nuclei were counterstained with DAPI (blue). Scale bars, 10 μm. **g** Representative images and quantification of UCP2 WT or KO PTCs treated with or without H/R with Oil red O staining. Scale bars, 10 μm. *p < 0.05, ANOVA.

UCP2 deficiency and corresponding TIF after I/R were associated with lipid accumulation in vivo. We investigated whether UCP2 regulates the production of ECM and accumulation of lipid in PTCs. We used an established model of I/R in vitro, the reoxygenation of hypoxia/reoxygenation-cultured PTCs (H/R). We analyzed ECM and lipids in PTCs isolated from UCP2 KO mice and corresponding WT mice. UCP2 KO PTCs displayed reduced expression (Fig. 3a) and extracellular deposition (Fig. 3b) of fibronectin and collagen I in response to H/R, relative to UCP2 WT PTCs. Importantly, UCP2 KO PTCs also displayed restored mRNA (Fig. 3c, d) and protein (Fig. 3e, f) expression of PPARα and CPT1α in response to H/R compared with UCP2 WT PTCs. Consistent with protein levels, UCP2 deficiency significantly reduced lipid droplets deposition after H/R (Fig. 3g). Overall, our results suggest that UCP2 regulates ECM production as well as accumulation of lipids in tubular cells.

### UCP2 regulates lipid deposition and ECM accumulation through HIF-1α in PTCs

**Fig. 4:**
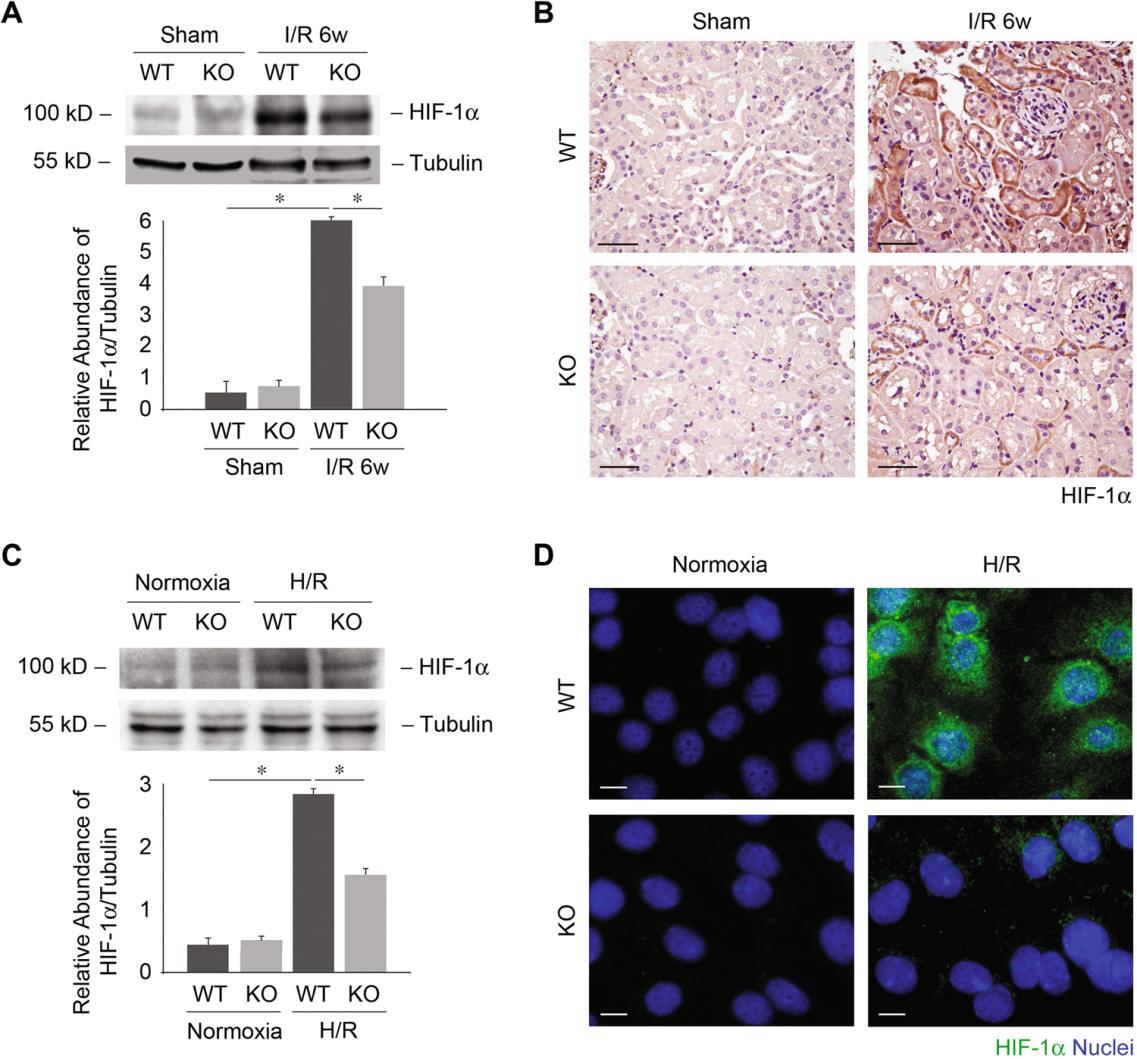
UCP2 regulates HIF-1α expression. **a** Immunoblot analysis for HIF-1α in kidney tissue from UCP2 WT or KO mice 6 weeks after I/R or sham laparotomy. Tubulin served as the standard. *p < 0.05, ANOVA. **b** Representative images of mice kidney sections from UCP2 WT or KO mice 6 weeks after I/R with immunostaining for HIF-1α. Scale bars, 50 μm. **c** Immunoblot analysis for HIF-1α of UCP2 WT or KO PTCs treated with or without H/R. Tubulin served as the standard. *p < 0.05, ANOVA. **d** Representative images of UCP2 WT or KO PTCs treated with or without H/R with immunostaining for HIF-1α (green). Nuclei were counterstained with DAPI (blue). Scale bars, 10 μm.

As described above, UCP2 deficiency was associated with fibrosis and lipids in vivo and in vitro, including reduced fibronectin and collagen I deposition, and restored PPARα and CPT1α expression (Figs. 2 and 3). ECM accumulations as well as PPARα and CPT1α expressions are regulated by HIF-1α^24–26^. We therefore evaluated whether UCP2 regulates HIF-1α. We first measured HIF-1α in kidney tissue. Increased expression of HIF-1α was observed in WT mice after I/R compared with those in sham-operated control mice. Meanwhile, KO mice displaced reduced HIF-1α relative to WT mice (Fig. 4a). Immune staining showed that increased HIF-1α mainly located in tubular cells of WT mice after I/R (Fig. 4b). Consistently, increased expression of HIF-1α was observed in UCP2 WT PTCs in response to H/R compared with those cultured in normoxia. While KO PTCs displaced reduced HIF-1α relative to WT PTCs (Fig. 4c). The increased HIF-1α mainly located in the nuclei of tubular cells (Fig. 4d). Overall, these results suggest that UCP2 regulates lipid deposition and ECM accumulation via HIF-1α in kidney tubule.

### UCP2 promotes the stabilization of HIF-1α through regulation of mitochondrial respiration and oxygen content in PTCs

**Fig. 5:**
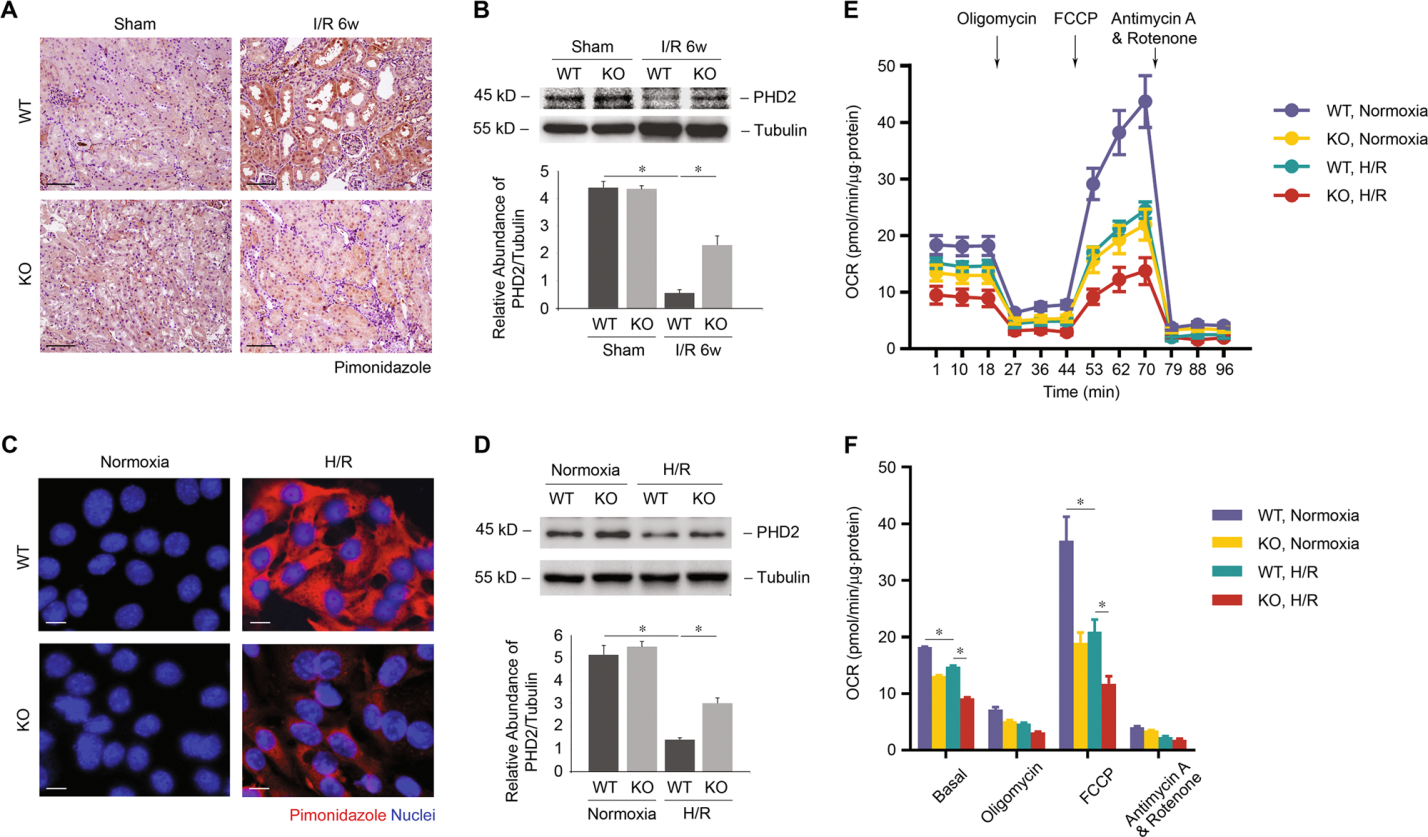
UCP2 promotes the stabilization of HIF-1α through regulation of mitochondrial respiration and oxygen content in tubular cells. **a** Representative images of mice kidney sections from UCP2 WT or KO mice 6 weeks after I/R with immunostaining for pimonidazole. Scale bars, 100 μm. **b** Immunoblot analysis for PHD2 in kidney tissue from UCP2 WT or KO mice 6 weeks after I/R or sham laparotomy. Tubulin served as the standard. *p < 0.05, ANOVA. **c** Representative images of UCP2 WT or KO PTCs treated with or without H/R with immunostaining for pimonidazole (red). Nuclei were counterstained with DAPI (blue). Scale bars, 10 μm. **d** Immunoblot analysis for PHD2 of UCP2 WT or KO PTCs treated with or without H/R. Tubulin served as the standard. *p < 0.05, ANOVA. **e** Representative traces show OCR in UCP2 WT or KO PTCs treated with or without H/R. Where indicted, oligomycin (1 μmol/L), FCCP (0.75 μmol/L), antimycin A, and rotenone (1 μmol/L) were added. **f** Summary data analyzed from three independent experiments shows OCR in UCP2 WT or KO PTCs treated with or without H/R. *p < 0.05, ANOVA.

HIF-1α is stabilized in response to hypoxia when prolyl hydroxylase domain (PHD) is inhibited^32,33^. Consistent with the stabilization of HIF-1α in renal tubule, pimonidazole staining, which indicates the decrease of oxygen content in tissue or cells, showed hypoxia in WT mice after I/R compared with those in sham-operated control mice. Meanwhile, KO mice displaced less hypoxia relative to WT mice (Fig. 5a). Decreased expression of PHD2 in WT mice after I/R compared with those in sham-operated control mice was restored in KO mice (Fig. 5b). In cultured PTCs, consistent with the above in vivo data, UCP2 KO PTCs also displayed less hypoxia in response to H/R compared with UCP2 WT PTCs (Fig. 5c). Decreased expression of PHD2 in WT PTCs in response to H/R compared with those cultured in normoxia was restored in KO PTCs (Fig. 5d). PTCs treated with H/R had lower baseline OCR and a reduction in FCCP-induced elevation in OCR, indicating low activity of mitochondrial respiration. The reduction in FCCP-induced elevation in OCR in WT PTCs was partially restored in KO PTCs (Fig. 5e, f). Taken together, these results suggest that UCP2 promotes the stabilization of HIF-1α via regulation of mitochondrial respiration and oxygen content in kidney tubular cells.

### HIF-1α regulates lipid deposition and ECM accumulation in PTCs

**Fig. 6:**
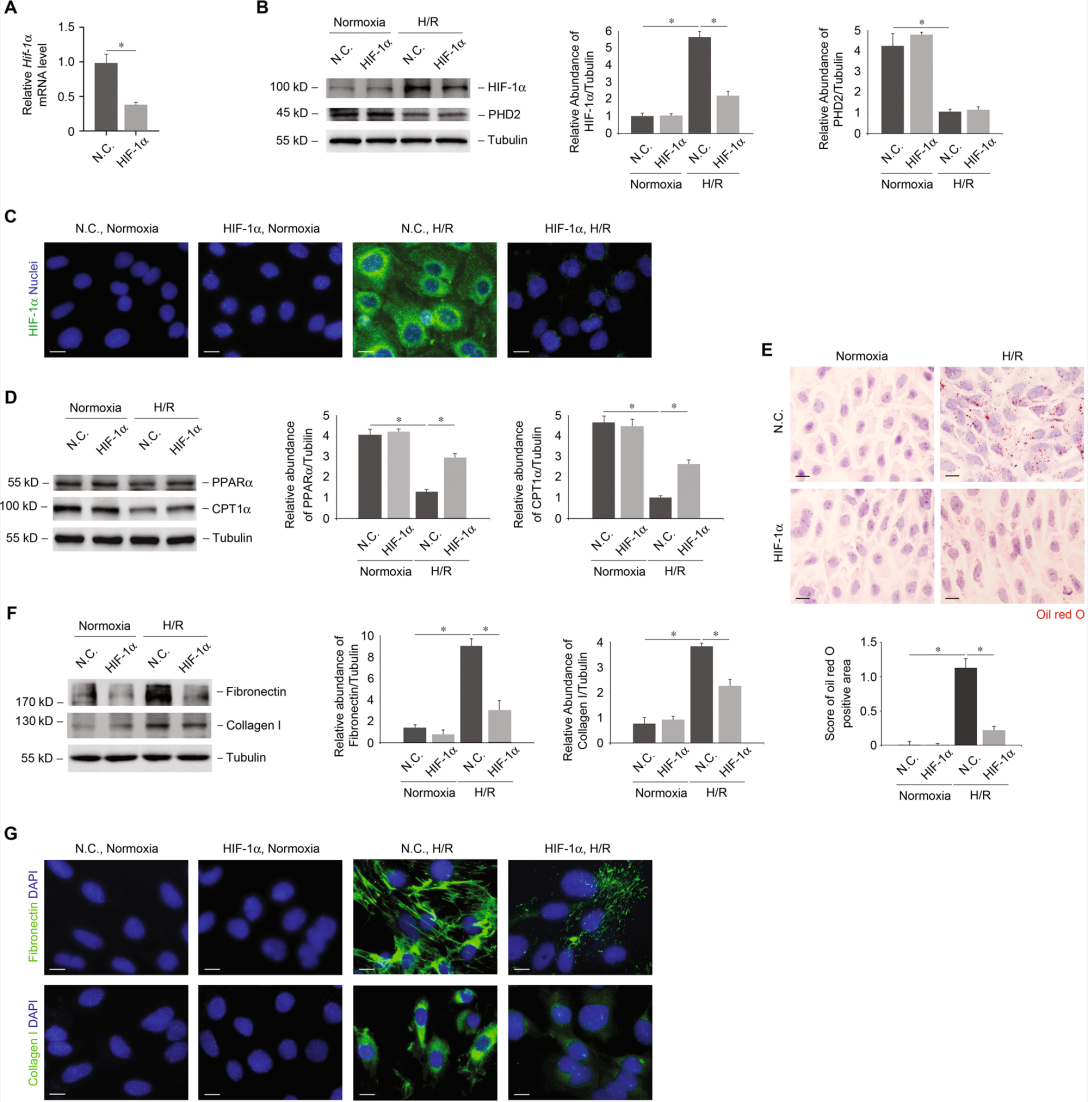
HIF-1α regulates lipid deposition and ECM accumulation in PTCs. **a** Relative mRNA levels of *Hif-1α* in *Hif-1α* siRNA or negative control (N.C.) siRNA-transducted PTCs treated with H/R; *n* = 3 per group. *p < 0.05, ANOVA. **b** Immunoblot analysis for HIF-1α and PHD2 of *Hif-1α* siRNA or N.C. siRNA-transducted PTCs treated with or without H/R. Tubulin served as the standard. *p < 0.05, ANOVA. **c** Representative images of *Hif-1α* siRNA or N.C. siRNA*-*transducted PTCs treated with or without H/R with immunostaining for HIF-1α (green). Nuclei were counterstained with DAPI (blue). Scale bars, 10 μm. **d** Immunoblot analysis for PPARα and CPT1α of *Hif-1α* siRNA or N.C. siRNA*-*transducted PTCs treated with or without H/R. Tubulin served as the standard. *p < 0.05, ANOVA. **e** Representative images and quantification of *Hif-1α* siRNA or N.C. siRNA-transducted PTCs treated with or without H/R with Oil red O staining. Scale bars, 10 μm. *p < 0.05, ANOVA. **f** Immunoblot analysis for fibronectin and collagen I of *Hif-1α* siRNA or N.C. siRNA-transducted PTCs treated with or without H/R. Tubulin served as the standard. *p < 0.05, ANOVA. **g** Representative images of *Hif-1α* siRNA or N.C. siRNA-transducted PTCs treated with or without H/R with immunostaining for fibronectin or collagen I (green). Nuclei were counterstained with DAPI (blue). Scale bars, 10 μm.

HIF-1α was preferentially downregulated in UCP2 KO renal tubule and cultured PTCs, in association with inhibition of lipid deposition and ECM accumulation. We therefore sought to investigate the role of HIF-1α in lipid and ECM regulation in PTCs. We analyzed whether deficiency of HIF-1α could suppress lipid droplets deposition and ECM accumulation in response to profibrosis stimuli. Genetic ablation of tubular epithelial *Hif-1α* inhibits the development of TIF in obstructive kidney, which is associated with decreased interstitial ECM deposition^23^. Here, we used RNA interference against mouse *Hif-1α* to knockdown HIF-1α in WT PTCs. The mRNA (Fig. 6a), protein (Fig. 6b), and nuclei translocation (Fig. 6c) of HIF-1α in response to H/R treatment was suppressed by *Hif-1α* siRNA transduction relative to N.C. siRNA transduction. Moreover, knockdown of HIF-1α rescued the protein expression of PPARα and CPT1α (Fig. 6d). Consistent with the results concerning the enzymes of lipid, knockdown of HIF-1α significantly suppressed the deposition of lipid droplets compared with N.C. (Fig. 6e). Similarly, knockdown of HIF-1α reduced the production (Fig. 6f) and accumulation (Fig. 6g) of fibronectin and collagen I in response to H/R treatment relative to N.C. Taken together, HIF-1α regulates lipid deposition and ECM accumulation in PTCs.

## Discussion

In this study, we demonstrate the physiological relevance of the mitochondrial protein UCP2 in mediating TIF in animal model of I/R-induced chronic renal fibrosis. We have shown that UCP2 acts as a critical regulator of lipid deposition and ECM accumulation in vivo and in vitro. We describe a molecular mechanism whereby HIF-1α, a critical factor that regulates lipid and ECM, serves as a downstream target molecule of UCP2-dependent oxygen content regulation. We show that UCP2-dependent lipid and ECM accumulation under the regulation HIF-1α is critical for TIF after ischemic injury. Similar to UCP2-deficient mice, genetic inhibition of HIF-1α rescued the reduction of PPARα and CPT1α, suppressed lipid droplets deposition and the synthesis, as well as accumulation of fibronectin and collagen I in vitro. Moreover, we describe a molecular mechanism by which UCP2 regulates HIF-1α stabilization through regulation of mitochondrial respiration and tissue hypoxia during TIF. Our current study suggests that UCP2 regulates TIF through the regulation of HIF-1α stabilization.

The kidney is one of the most energy-demanding organs that requires a large number of mitochondria to remove waste, and regulate fluid and electrolyte balance. PTs require more active transport mechanisms than other renal cell types because they reabsorb 80% of the filtrate that passes through the glomerulus. As such, they contain more mitochondria than other structure in the kidney. Maintaining mitochondrial homeostasis is critical to the proper functioning of the PTs. Previous studies show that persistent mitochondrial dysfunction is evident in the early stages and progression of renal disease^1,2^. UCP2 resides in the inner membrane of mitochondria from where energy is provided through OXPHOS process. Loss of UCP2 attenuates mitochondrial dysfunction without altering ROS production and uncoupling activity^34^, probably suggests a weak uncoupling activity of UCP2. Besides the regulation of energy production via uncoupling OXPHOS from ATP synthesis with energy dissipation^4–6^, UCP2 has also been shown to be involved in numerous pathophysiologic conditions commonly observed in various kidney diseases, including metabolic disorders^35^, inflammation^11^, ischemic injury^28^, and fibrosis^17^. Our human and mice data suggest that UCP2 expression was increased in tubule with profibrosis injury as previously reported^17^. Moreover, tubular cell-specific UCP2 deficiency reduces I/R-induced chronic TIF, suggesting that UCP2 was critical in the progression of fibrosis. Our previous study shows that global KO of UCP2 suppresses the production of ECM in obstructive kidney; however, the mechanism remains elusive. Nevertheless, UCP2 shows beneficial effects via protection of cellular autophagy and mitochondrial dynamics, and reduction of apoptosis of tubular cells in acute ischemic kidney injury^18,28^. The dual effect of UCP2 may be explained by the distinct pathophysiologic alternations in acute and chronic kidney injury. As compared to apoptosis and death in acute injury, tubular cells in chronic kidney injury are characterized by changes, such as synthesis of ECM. The resulted histological differences are also significant, with acute tubular loss and proliferation vs. chronic tubular atrophy and fibrosis. Therefore, the timing and approach for UCP2-targeting treatment need further investigations.

Tubular epithelial lipid accumulation has received substantial attention in the context of kidney diseases. Metabolism of lipid requires their transport into the mitochondria mediated by CPT1α, which is considered to be the rate-limiting enzyme in lipid catabolism. The PPARα is key transcription factor that regulates the expressions of proteins involved in lipid metabolism. The association of lipid metabolism and renal fibrosis has been demonstrated in human and mouse with fibrosis, and agonists of PPARα and CPT1α have better therapeutic profile^19^. UCP2 and lipid is interconnected. UCP2 can bind fatty acid laterally through its peripheral site, and this intramembrane molecular recognition is essential for UCP2-catalyzed fatty acid flipping across the membrane, which in turn is essential for proton translocation^36^. Elevated expression of UCP2 was in accordance with increased intracellular free fatty acids^37^ and decreased lipolysis^8^. UCP2 promotes lipid synthesis in macrophage^11^. UCP2 prevents mitochondrial oxidation and promotes stem cells proliferation^16^. In cancer cells, UCP2 regulates fatty acid oxidation and mitochondrial respiration, as well as tumorigenic properties^12,13^. These results provide evidence that UCP2 has a significant regulatory role in lipid and cellular metabolism. Our results show that UCP2 regulates cellular lipid deposition during TIF. Furthermore, our data show that the UCP2-mediated profibrotic phenotype is regulated by HIF-1α-dependent lipid and ECM accumulation. UCP2 KO PTCs displayed reduced lipid droplets deposition, as well as production and accumulation of ECM as a result of HIF-1α downregulation. Consistent with the results observed in UCP2 KO cells, inhibition of HIF-1α also suppressed the accumulation of lipid and ECM in PTCs. Although the functions of UCP2 in different tissue types and different pathologic processes remain controversial, our results suggest that UCP2 has a significant impact on lipid metabolic processes in tubular cells.

Hypoxia and dysregulated metabolism are first and foremost defining features of tumors^38^. In the past two decades, hypoxia has been proposed as an important microenvironment factor in the development of renal fibrosis^39–41^. The critical mediator of cellular adaptation to hypoxia is HIF. Clinical and genetic evidence show that activation of HIF signaling in renal epithelial cells is associated with the development of chronic renal fibrosis and may promote expression and accumulation of ECM^23,42,43^. Recent studies demonstrated that HIF-1α regulates lipid metabolism and the involved key regulator and enzymes^24–26^. The functions of HIF-1α in acute and CKDs seem to diametrically distinct^20,22,44^. Consistent with previous studies concerning renal fibrosis, our results show that inhibition of HIF-1α rescued the reduction of PPARα and CPT1α, suppressed lipid droplets deposition and the synthesis, as well as accumulation of fibronectin and collagen in PTCs, suggest that UCP2-dependent lipid and ECM accumulation is regulated by HIF-1α. Under hypoxia, two critical prolyl residues of HIF-α are hydroxylated by specific PHD-containing proteins and rapid degradation by proteasome^32,33^. All PHDs (PHD1, PHD2, and PHD3) are expressed in renal tubular epithelial cells^45^. However, PHD2 is a key regulator of HIF-α expression^46^. Our in vivo and in vitro data suggest that UCP2 deficiency attenuates hypoxia by modulation of mitochondrial respiration and oxygen content in PTCs and therefore restores PHD2 mediated HIF-1α degradation.

In conclusion, our data suggest that UCP2 regulates TIF through lipid deposition and ECM accumulation mediated by HIF-1α. Also, our data describe a molecular mechanism by which UCP2 regulates HIF-1α stabilization through regulation of mitochondrial respiration and tissue hypoxia during TIF. Further investigations may be useful for identify UCP2 as a potential therapeutic target in treating chronic renal fibrosis.

## References

[1] Bhargava P, Schnellmann RG (2017). Mitochondrial energetics in the kidney. Nat. Rev. Nephrol..

[2] Galvan DL, Green NH, Danesh FR (2017). The hallmarks of mitochondrial dysfunction in chronic kidney disease. Kidney Int..

[3] Tang C, Dong Z (2016). Mitochondria in kidney injury: when the power plant fails. J. Am. Soc. Nephrol..

[4] Arsenijevic D (2000). Disruption of the uncoupling protein-2 gene in mice reveals a role in immunity and reactive oxygen species production. Nat. Genet..

[5] Fleury C (1997). Uncoupling protein-2: a novel gene linked to obesity and hyperinsulinemia. Nat. Genet..

[6] Rousset S (2004). The biology of mitochondrial uncoupling proteins. Diabetes.

[7] Andrews ZB (2005). Uncoupling protein-2 is critical for nigral dopamine cell survival in a mouse model of Parkinson’s disease. J. Neurosci..

[8] Anedda A, Rial E, Gonzalez-Barroso MM (2008). Metformin induces oxidative stress in white adipocytes and raises uncoupling protein 2 levels. J. Endocrinol..

[9] Ayyasamy V (2011). Cellular model of Warburg effect identifies tumor promoting function of UCP2 in breast cancer and its suppression by genipin. PLoS ONE.

[10] Diano S (2003). Uncoupling protein 2 prevents neuronal death including that occurring during seizures: a mechanism for preconditioning. Endocrinology.

[11] Moon JS (2015). UCP2-induced fatty acid synthase promotes NLRP3 inflammasome activation during sepsis. J. Clin. Invest..

[12] Pecqueur C (2008). Uncoupling protein-2 controls proliferation by promoting fatty acid oxidation and limiting glycolysis-derived pyruvate utilization. Faseb. J..

[13] Samudio I, Fiegl M, McQueen T, Clise-Dwyer K, Andreeff M (2008). The warburg effect in leukemia-stroma cocultures is mediated by mitochondrial uncoupling associated with uncoupling protein 2 activation. Cancer Res..

[14] Sheets AR (2008). Uncoupling protein-2 modulates the lipid metabolic response to fasting in mice. Am. J. Physiol. Gastrointest. Liver Physiol..

[15] Vozza A (2014). UCP2 transports C4 metabolites out of mitochondria, regulating glucose and glutamine oxidation. Proc. Natl Acad. Sci. USA.

[16] Zhang J (2011). UCP2 regulates energy metabolism and differentiation potential of human pluripotent stem cells. Embo. J..

[17] Jiang L (2013). A microRNA-30e/mitochondrial uncoupling protein 2 axis mediates TGF-beta1-induced tubular epithelial cell extracellular matrix production and kidney fibrosis. Kidney Int..

[18] Qin N (2019). UCP2-dependent improvement of mitochondrial dynamics protects against acute kidney injury. J. Pathol..

[19] Kang HM (2015). Defective fatty acid oxidation in renal tubular epithelial cells has a key role in kidney fibrosis development. Nat. Med..

[20] Haase VH (2006). The VHL/HIF oxygen-sensing pathway and its relevance to kidney disease. Kidney Int..

[21] Li L (2019). FoxO3 activation in hypoxic tubules prevents chronic kidney disease. J. Clin. Invest..

[22] Shu S (2019). Hypoxia and hpoxia-inducible factors in kidney injury and repair. Cells.

[23] Higgins DF (2007). Hypoxia promotes fibrogenesis in vivo via HIF-1 stimulation of epithelial-to-mesenchymal transition. J. Clin. Invest..

[24] Du W (2017). HIF drives lipid deposition and cancer in ccRCC via repression of fatty acid metabolism. Nat. Commun..

[25] Huang (2014). HIF-1-mediated suppression of acyl-CoA dehydrogenases and fatty acid oxidation is critical for cancer progression. Cell Rep..

[26] Narravula S, Colgan SP (2001). Hypoxia-inducible factor 1-mediated inhibition of peroxisome proliferator-activated receptor alpha expression during hypoxia. J. Immunol..

[27] Kumar S (2009). Dexamethasone ameliorates renal ischemia-reperfusion injury. J. Am. Soc. Nephrol..

[28] Zhou Y (2017). UCP2 attenuates apoptosis of tubular epithelial cells in renal ischemia-reperfusion injury. Am. J. Physiol. Ren. Physiol..

[29] Terryn S (2007). A primary culture of mouse proximal tubular cells, established on collagen-coated membranes. Am. J. Physiol. Ren. Physiol..

[30] Jiang L (2013). Rheb/mTORC1 signaling promotes kidney fibroblast activation and fibrosis. J. Am. Soc. Nephrol..

[31] Yang J, Liu Y (2001). Dissection of key events in tubular epithelial to myofibroblast transition and its implications in renal interstitial fibrosis. Am. J. Pathol..

[32] Jaakkola P (2001). Targeting of HIF-alpha to the von Hippel-Lindau ubiquitylation complex by O2-regulated prolyl hydroxylation. Science.

[33] Semenza GL (2011). Oxygen sensing, homeostasis, and disease. N. Engl. J. Med..

[34] Kukat A (2014). Loss of UCP2 attenuates mitochondrial dysfunction without altering ROS production and uncoupling activity. PLoS Genet..

[35] Qiu W (2012). Genipin inhibits mitochondrial uncoupling protein 2 expression and ameliorates podocyte injury in diabetic mice. PLoS ONE.

[36] Berardi MJ, Chou JJ (2014). Fatty acid flippase activity of UCP2 is essential for its proton transport in mitochondria. Cell Metab..

[37] Xu H (2015). Uncoupling lipid metabolism from inflammation through fatty acid binding protein-dependent expression of UCP2. Mol. Cell Biol..

[38] Xie H, Simon MC (2017). Oxygen availability and metabolic reprogramming in cancer. J. Biol. Chem..

[39] Manotham K (2004). Evidence of tubular hypoxia in the early phase in the remnant kidney model. J. Am. Soc. Nephrol..

[40] Norman JT, Clark IM, Garcia PL (2000). Hypoxia promotes fibrogenesis in human renal fibroblasts. Kidney Int..

[41] Orphanides C, Fine LG, Norman JT (1997). Hypoxia stimulates proximal tubular cell matrix production via a TGF-beta1-independent mechanism. Kidney Int..

[42] Kimura K (2008). Stable expression of HIF-1alpha in tubular epithelial cells promotes interstitial fibrosis. Am. J. Physiol. Ren. Physiol..

[43] Stegen S (2019). HIF-1alpha metabolically controls collagen synthesis and modification in chondrocytes. Nature.

[44] Weidemann A (2008). HIF activation protects from acute kidney injury. J. Am. Soc. Nephrol..

[45] Soilleux EJ (2005). Use of novel monoclonal antibodies to determine the expression and distribution of the hypoxia regulatory factors PHD-1, PHD-2, PHD-3 and FIH in normal and neoplastic human tissues. Histopathology.

[46] Berra E (2003). HIF prolyl-hydroxylase 2 is the key oxygen sensor setting low steady-state levels of HIF-1alpha in normoxia. Embo. J..

